# Comparative evaluation of four hydrogen peroxide-based systems to decontaminate N95 respirators

**DOI:** 10.1017/ash.2021.183

**Published:** 2021-08-18

**Authors:** Shawn Clark, Herman Ng, Ge Wu, Devika Jain, Garry Bassi, Rita Kandel, Tony Mazzulli

**Affiliations:** 1 Department of Laboratory Medicine and Pathobiology, University of Toronto, Toronto Canada; 2 Department of Microbiology, University Health Network/Sinai Health System, Toronto Canada; 3 Medical Engineering, University Health Network, Toronto Canada; 4 Medical Device Reprocessing, Mount Sinai Hospital, Toronto Canada; 5 Department of Pathology and Laboratory Medicine, Mount Sinai Hospital, Toronto Canada

## Abstract

**Objective:**

Protocols designed to facilitate N95 filtering facepiece respirator (FFR) decontamination by commercial sterilization devices do not recommend that operators verify the device’s performance against pathogens deposited on FFRs. Here, we compared the treatment efficacy of 4 hydrogen peroxide-based systems that were authorized for N95 decontamination during the COVID-19 pandemic.

**Methods:**

Suspensions prepared from *S. aureus* ATCC 29213 and 44300, *B. subtilis* ATCC 6633, a vancomycin-resistant *E. faecium* isolate (VRE), *E. coli* ATCC 25922, and *P. aeruginosa* ATCC 27853 colonies were inoculated onto nine 1-cm^2^ areas on a 3M 1805, 1860, 1860S, 1870+, 8210, 8110S, or 9105S FFR. Contaminated respirators were treated according to protocols recommended by the STERRAD 100NX, Bioquell Z-2, Sterizone VP4, or Clēan Works Mini systems. Decontamination efficacy was determined by comparing colony counts cultured from excised segments of treated and untreated FFR.

**Results:**

All devices achieved a 6-log reduction in bacterial burden and met FDA sterilization criteria. The Bioquell Z-2 device demonstrated 100% efficacy against both gram-positive and gram-negative organisms with all FFRs tested. Colonies of *S. aureus* ATCC 29213 and 44300 and VRE were cultivable from up to 9 (100%) of 9 STERRAD 100NX– and Sterizone VP4–treated segments. Viable *B. subtilis* ATCC 6633 organisms were recovered from 76.0% of STERRAD 100NX–treated FFR segments.

**Conclusions:**

Variability in decontamination efficacy was noted across devices and FFR types. gram-positive organisms were more difficult to completely eliminate than were gram-negative organisms. Prior to initiating FFR decontamination practices, institutions should verify the effectiveness of their devices and the safety of treated FFR.

The emergence and spread of the novel human coronavirus severe acute respiratory syndrome coronavirus 2 (SARS-CoV-2) has placed significant strain on healthcare resources. Single-use N95 filtering facepiece respirators (FFRs) are essential tools in the prevention of SARS-CoV-2 transmission among those in patient-facing roles.^1^ An overwhelming demand for ventilators, laboratory consumables, and personal protective equipment (PPE), including FFR, has created shortages that were further affected by the disruption of global supply chains.^2^ In response, many healthcare institutions designed tiered plans for PPE inventory monitoring similar to those recommended by the Centers for Disease Control and Prevention.^3,4^ These measures included allowing FFR to be worn for multiple patient contacts, limiting their use to specific clinical situations (eg, aerosol-generating procedures) or reprocessing and reusing N95 FFR beyond their shelf life.

The reprocessing of FFR involves a decontamination procedure to remove bioburden from the FFR surface that may include infectious particles (eg, bacteria, viruses, or fungi). This can be achieved using systems designed for the sterilization of medical devices or those used for postdischarge decontamination of hospital rooms.^5^ Hydrogen peroxide (H_2_O_2_), ultraviolet germicidal irradiation, and moist and dry heat methods have been examined for this purpose.^6–9^ To ensure that these treatments do not compromise the FFR, filtration, fit and any chemical residue must be evaluated in accordance with industry standards.^10,11^ Several commercial H_2_O_2_-based systems have received emergency authorization for FFR decontamination during the coronavirus disease (COVID-19) pandemic due to their minimal impact on respirator integrity.^12,13^ Multiple healthcare facilities in North America have openly discussed the design and implementation of their FFR reprocessing during the COVID-19 pandemic^5,14–16^; however, none have described evaluations of the efficacy of their processes through sterility testing or mechanisms for continuous quality monitoring.

The concept of FFR decontamination and reuse is not unique to the COVID-19 pandemic,^17,18^ but it remains somewhat controversial. The utility of these processes relies on their ability to reduce or eliminate pathogens and other biological substances from porous FFR material. Previously worn N95 FFR can retain high burdens of live microorganisms, which may be present alongside other biological materials that can interfere with the decontamination process.^5,19^ Given the current pandemic, many recent studies focus primarily on the activity of H_2_O_2_-based methods against SARS-CoV-2 or surrogate viruses. However, some patients infected with SARS-CoV-2 can have co-occurring or secondary bacterial infections.^20^ This finding suggests that aerosols or other respiratory fluids may be polymicrobial and could include organisms that are more resistant to H_2_O_2_ treatment. The efficacies of H_2_O_2_ decontamination processes against nonviral organisms have not been fully evaluated. In this study, we evaluated the efficacy of 4 H_2_O_2_-based decontamination methods to reduce gram-positive and gram-negative loads on artificially contaminated N95 FFR.

## Materials and methods

### Bacterial strains and culture conditions

To represent bacteria that vary in their resistance to hydrogen peroxide, we selected 4 gram-positive bacteria: *Staphylococcus aureus* ATCC 29213 (methicillin susceptible, MSSA), *S. aureus* ATCC 43300 (methicillin resistant, MRSA), *Bacillus subtilis* ATCC 6633, and a vancomycin resistant clinical isolate of *Enterococcus faecium*. We then selected 2 gram-negative bacteria: *Escherichia coli* ATCC 25922, *Pseudomonas aeruginosa* ATCC 27853. Each organism was freshly subcultured onto Columbia agar with 5% (v/v) sheep blood (Thermo Fisher Scientific, Nepean, ON, Canada) and incubated in 5% CO_2_ at 35°C for 18–24 hours prior to each experiment.

### Decontamination devices, respirator inoculation, and processing

We evaluated 4 H_2_O_2_-based decontamination devices that had been used to treat N95 FFRs at 4 clinical centers across the Greater Toronto Area (Ontario, Canada) during the first COVID-19 pandemic wave: STERRAD 100NX (Advanced Sterilization Products, Irvine, CA), Sterizone VP4 (Stryker, Kalamazoo, MI), Clēan Works Clean Flow Mini (Clean Works, St Catherine’s, ON, Canada) and Bioquell Z-2 (EcoLab, St Paul, MN) systems (Table 1). Devices were operated in accordance with recommended N95 FFR decontamination protocols (Table 1).

**Table 1.** Differences in N95 Decontamination Parameters Across the 4 Devices Used

Device	Modality	Capacity^a^	Processed in Tyvek Pouch	Treatment conditions^b^	H_2_O_2_ %
Bioquell Z-2 (EcoLab Life Sciences)	Vaporized hydrogen peroxide	Depends on room size (≥100)	No	Conditioning: 12 min Gassing: 85 min (10.0 g/min HPV peak 349 ppm) Aeration: 107 min	30.0
Clēan Flow Mini (Clean Works)	Ultraviolet-C, hydrogen peroxide, ozone hybrid	>100	No	Standard procedure Gassing: 40 mL/min UV-C dose: 59 mJ/cm^2^ Ozone: 30 s	3.0
Sterizone VP4 (Stryker)	Vaporized hydrogen peroxide-ozone hybrid	15–20	Yes	N95 respirator decontamination cycle Gassing: 3.5 min	50.0
STERRAD 100NX (Advanced Sterilization Products)	Hydrogen peroxide gas plasma	15–20	Yes	Express cycle Time: 24 min Loading: Bottom shelf Cycle temperature: 47–56°C	58.0

Note. HPV, hydrogen peroxide vapor; UV-C, ultraviolet C light.

a Reflects the total capacity of a device suggested by the manufacturer under the assumption that no additional factors (eg, staffing) are limiting factors.

b As recommended by the respective manufacturer.

Devices were challenged with up to 5 different 3M N95 FFR models (3M 1860, 1860S, Aura 1870+, 8210, 8110S, or VFlex 9105S or 1805) that were cut in half to conserve FFR supplies. Briefly, 3 cell suspensions of each test organism equivalent to a 0.5 McFarland standard (˜1.5×10^8^ cells/mL) were prepared by emulsifying 4–5 colonies from fresh subculture plates into sterile 0.45% (v/v) saline. Three 1-cm × 1-cm squares were drawn onto the patient-facing side of each FFR half using a permanent marker and inoculated with 10 µL of the corresponding 0.5 McFarland suspension. Five FFR halves were inoculated for each device, which included a positive (untreated) and negative (inoculated with 0.45% (v/v) saline) control. Following inoculations, FFRs were air dried (20–30 minutes) and placed into a sealed Tyvek pouch for distribution to the testing sites. Decontamination was performed according to the device manufacturer’s recommendations (Table 1). Successful cycle completion was confirmed using biological monitors or indicators where appropriate. Respirators treated by the Bioquell Z-2 and Clean Works instruments were removed from their packaging and repackaged after treatment. All FFRs were returned to the University Health Network/Sinai Health Microbiology Laboratory (Toronto, ON) within 24 hours. Additional procedural information can be found in the Supplementary Materials (online).

### Determination of decontamination efficacy

Inoculated segments (1-cm^2^ squares) were excised from treated FFR using sterile scissors and placed into 10 mL brain heart infusion broth (Thermo Fisher). Colony counts were obtained as follows: inoculated tubes were placed in an orbital shaker (4°C for 5 minutes at 250 rpm), mixed with a vortexer (5 seconds), placed in 1-mL aliquots on a Columbia sheep blood agar plate, and incubated for 18–24 hours at 37°C in 5% CO_2_. The remaining broth was incubated (48 hours at 37°C) and subcultured onto Columbia sheep blood agar if turbid. Colonies were identified using the VITEK matrix-assisted laser desorption/ionization time-of-flight mass spectroscopy (MALDI-TOF MS) system (bioMérieux, Marcy-l'Étoile, France). Decontamination efficacy, defined as the percentage reduction in colony-forming units (CFU), was determined by comparing colony counts cultured from treated and untreated FFR segments. The effectiveness of sterilization was determined using Health Canada guidance (e.g. minimum 6-log reduction) (FDA 2020).^21^

## Results

Bacterial recovery was not influenced by FFR model because all test organisms could be cultured from excised FFR segments that had been inoculated, dried, and placed into a Tyvek pouch but not sterilized in initial pilot experiments (data not shown). All decontamination cycles in each instrument were completed successfully, as noted by biological monitors used at each testing site. All decontamination devices exceeded FDA recommendations (≥6-log reduction in the inoculated microbial burden for all 6 organisms based on an average inoculum (CFU/mL) of the bacterial suspension of 5.9×10^8^ for *E. coli* ATCC 25922, 6.5×10^8^ for *P. aeruginosa* ATCC 27853, 8.1×10^8^ for *S. aureus* ATCC 25923, 7.9*×*10^8^ for *S. aureus* ATCC 43300, 5.0×10^8^ for *B. subtilis* ATCC 6633, and 5.9×10^8^ for the clinical VRE isolate. However, complete sterilization of the N95 FFRs of all gram-positive organisms was not universally achieved.

All 4 systems performed equally well with gram-negative organisms. Neither *E. coli* ATCC 25922 nor *P. aeruginosa* ATCC 27853 were recovered on any of the inoculated segments from treated FFRs, corresponding to a decontamination efficacy of 100.0% and complete sterilization being achieved. The most effective system for decontamination in our hands was the Bioquell Z-2, which demonstrated 100% effectiveness and complete sterilization of all 6 test organisms on the five 3M FFR models tested (Table 2). Similar performance characteristics were observed with the Clēan Flow Mini system with no viable organisms being recovered for 5 of the 6 pathogens following decontamination. Nevertheless, this system had difficulty eliminating VRE, which was recovered on a single coupon from an 1805 model FFR.

**Table 2.** Comparative Evaluation of Bacterial Recovery From N95 FFRs Following Decontamination

Devices	Organisms	No. FFR Segments With Viable Bacteria (n=9 Replicates Each), No. (%)^a^							
		1805	8210	1860	1870	1860S	1870+	9105S^b^	8110S
STERRAD 100NX	MSSA	nt	9 (100)	9 (100)	nt	4 (44.4)	4 (44.4)	4 (44.4)	nt
	MRSA	nt	9 (100)	6 (66.6)	nt	3 (33.3)	6 (66.6)	4 (44.4)	nt
	VRE	nt	9 (100)	8 (88.9)	nt	6 (66.6)	3 (33.3)	5 (55.6)	nt
	*B. subtilis*	nt	9 (100)	8 (88.9)	nt	3 (33.3)	9 (100)	5 (55.6)	nt
	*E. coli*	nt	0	0	nt	0	0	0	nt
	*P. aeruginosa*	nt	0	0	nt	0	0	0	nt
Bioquell Z-2	MSSA	nt	0	nt	0	0	0	nt	0
	MRSA	nt	0	nt	0	0	0	nt	0
	VRE	nt	0	nt	0	0	0	nt	0
	*B. subtilis*	nt	0	nt	0	0	0	nt	0
	*E. coli*	nt	0	nt	0	0	0	nt	0
	*P. aeruginosa*	nt	0	nt	0	0	0	nt	0
Sterizone VP4	MSSA	0	0	nt	nt	0	2 (22.2)	nt	nt
	MRSA	3 (33.3)	0	nt	nt	0	0	nt	nt
	VRE	1 (11.1)	0	nt	nt	7 (77.8)	6 (66.6)	nt	nt
	*B. subtilis*	0	0	nt	nt	0	0	nt	nt
	*E. coli*	1 (11.1)	0	nt	nt	0	0	nt	nt
	*P. aeruginosa*	0	0	nt	nt	0	0	nt	nt
Clēan Flow Mini	MSSA	0	0	nt	nt	0	0	nt	nt
	MRSA	0	0	nt	nt	0	0	nt	nt
	VRE	1 (11.1)	0	nt	nt	0	0	nt	nt
	*B. subtilis*	0	0	nt	nt	0	0	nt	nt
	*E. coli*	0	0	nt	nt	0	0	nt	nt
	*P. aeruginosa*	0	0	nt	nt	0	0	nt	nt

a nt, not tested.

b Cellulose containing respirator and should not be decontaminated with H_2_O_2_.

Although acceptable CFU reductions based on FDA decontamination standards, the H_2_O_2_ gas plasma STERRAD 100NX and H_2_O_2_/ozone Sterizone VP4 systems were unable to completely sterilize FFR inoculated with gram-positive bacteria (Table 2). Both MSSA and MRSA strains remained viable on up to 100.0% of FFR segments following decontamination by the STERRAD 100NX, with mean percentage reductions of 99.97% (range, 99.84%–100.0%) and 99.98% (range, 99.91%–100%), respectively. The *B. subtilis* strain was completely sterilized in only 11 of the 45 total excised segments (24.4%) from STERRAD 100NX–treated FFR, with an average of 11.2 CFU (range, 2–46 CFUs) recovered from the remaining inoculated segments posttreatment (data not shown). Similar to the *S. aureus* strains, VRE remained viable on up to 100.0% of the inoculated segments (range, 33.3%–100.0%), with mean percentage reductions in CFU of 99.96% to 99.99%. The 3M 8210 model FFR performed the poorest with the STERRAD 100NX treatment, and all 4 gram-positive organisms were recovered from all 36 segments inoculated (100.0%). In contrast, the Sterizone VP4 system performed well with MSSA and *B. subtilis* (100.0% reduction). An average of 64 CFU of MRSA were recovered from 4 of 36 inoculated segments, most of which (75.0%) were extracted from the 1805 FFR model. The VRE strain was 100.0% killed following sterilization in 21 of 36 inoculated segments tested, and the percentage reduction ranged from 99.86% to 99.99% in the remaining segments. In contrast to the performance in the STERRAD 100NX, no viable organisms were recovered from the 3M 8210 FFR following treatment with the Sterizone VP4 device.

## Discussion

The implementation of FFR decontamination and reuse strategies can assist healthcare facilities with restoring depleted supplies to protect those in patient-facing roles from pathogens in droplets or aerosols. The 4 H_2_O_2_-based methods evaluated here differed in their ability to decontaminate artificially soiled FFR and were less likely to completely sterilize FFR carrying a high inoculum of gram-positive pathogens.

We challenged the 4 devices with bacteria that are more tolerant to H_2_O_2_ than other organisms such as enveloped viruses (eg, SARS-CoV-2).^7,22^ The properties of the 4 systems, the organisms tested, and FFR type can influence performance. Viable gram-positive organisms were recovered from FFR segments treated by the STERRAD 100NX and Sterizone VP4 systems. Differences in the physiologic properties of our test bacteria including cell-wall composition, organism size, and the production of environment-modulating enzymes, may contribute greatly to these distinct reactions. We inoculated FFRs with a high bacterial burden to simulate clinical situations such as pneumonia, in which secretions may contain upwards of 10^7^ CFU per gram of sputum.^23^ At high cell densities, H_2_O_2_ degradation can occur via catalase enzymes produced by *S. aureus* soon after exposure and can contribute to their persistence.^24^ Recently, Ibáñez-Cervantes et al^6^ reported that *S. aureus* was not recoverable from 3M 8210 model FFRs when inoculated at concentrations of 10^2^–10^6^ CFU and treated by the STERRAD NX system, which is discordant with our study. This incongruity may be explained by differences in cycling parameters between the STERRAD NX and 100NX systems, which also complicated our ability to compare results between studies.^25^

The Bioquell Z-2 device performed the best of the 4 devices in our study. This system was operated in a specially designed room dedicated to contactless sterilization, and stringent parameters were applied to prevent the dissipation of sterilant and ensure optimal distribution and contact (eg, sterilization-resistant surfaces and barriers). Wigginton et al^7^ noted that *S. aureus* ATCC 29213 could be recovered with high frequency from inoculated 3M 1860 FFR segments following treatment with a similar Bioquell Q10 system, which also performed poorly with several viruses.^7^ Whether differences in experimental design played a role in these variable results is unclear. Challenges with FFR decontamination by low-temperature sterilization approaches are perhaps not surprising; recent reports have noted sterilization failures with *S. aureus*, VRE, and other organisms on other nonporous surfaces such as stainless steel.^19,26^ Thus, additional sterility monitoring (alongside any biological test standards) should be used to ensure the safety and efficacy of these processes among decontaminated FFR batches, particularly when FFR have been previously treated and reused.

Our study has several limitations. FFR resources were limited, so only a small number were processed in this study and all were cut in half for testing. We did not assess variation in decontamination efficacy across different parts of the FFRs (eg, the head straps or metal nosepiece) or the impact of the 4 sterilization modalities on fit, filtration efficiency, and any potential posttreatment chemical exposures to healthcare providers. Our test organisms were limited to several common bacterial pathogens with varying levels of H_2_O_2_ resistance due to the enhanced biosafety requirements needed to propagate SARS-CoV-2 and other viruses. Because FFRs were inoculated directly from cell suspensions rather than in the presence of a biological sample matrix that may impair decontamination efficacy (eg, saliva, sputum, or pus), the results of our evaluation may not translate directly to the clinical setting. Regardless, we demonstrated that gram-positive organisms were difficult to decontaminate the H_2_O_2_ treated FFRs.

Reductions in CFU equivalent to 6-log was noted for all test organisms, which may be insufficient in heavily contaminated FFR where the starting bacterial load may be much higher than that used here. The persistence of epidemiologically significant organisms, such as MRSA and VRE, in the hospital environment is of concern due to the possible spread, colonization, and safety risk this may pose to both patients and healthcare providers. There is some debate regarding whether our test organisms could be found on N95 FFR prior to decontamination. Hospital air sampling studies have identified MRSA and carbapenemase-producing *P. aeruginosa* among others, which suggests that these organisms could unknowingly contaminate FFR surfaces.^27,28^ Facilities must recognize the limitations associated with decontaminating and reusing FFRs that may have been soiled with clinically significant pathogens other than SARS-CoV-2.

In conclusion, prior to initiating FFR decontamination practices, institutions should ensure that quality assurance plans include assessments of FFR bioburden reduction and routine sterility monitoring to verify the effectiveness of decontamination and safety of reprocessed FFRs.

## Supplementary material

For supplementary material accompanying this paper visit https://doi.org/10.1017/ash.2021.183. click here to view supplementary material
