# Supplementary material for: Comparative evaluation of four hydrogen peroxide-based systems to decontaminate N95 respirators
Source: Antimicrob Steward Healthc Epidemiol. 2021 Aug 18;1(1):e21. doi: 10.1017/ash.2021.183 (PMC9495415; doi:10.1017/ash.2021.183)
Supplement: Supplementary file 1 [file S2732494X21001832sup001.docx]

**Supplementary Materials for

Comparative evaluation of four hydrogen peroxide-based systems to decontaminate N95 respirators**
Shawn T Clark, Herman Ng, Ge Wu, Devika Jain, Garry Bassi, Rita Kandel, Tony Mazzulli

**MATERIALS AND METHODS**

***Bacterial strains and culture conditions***

Six bacteria were selected for this evaluation due to their clinical and infection control significance. This included four Gram-positive bacteria, *Staphylococcus aureus* ATCC 29213 (methicillin susceptible, MSSA), *S. aureus* ATCC 43300 (methicillin resistant, MRSA), *Bacillus subtilis* ATCC 6633, and a clinical isolate of *Enterococcus faecium* (vancomycin resistant) and two Gram-negative bacteria, *Escherichia coli* ATCC 25922, *Pseudomonas aeruginosa* ATCC 27853. Prior to the experiments, a fresh subculture of each microorganism was streaked onto Columbia agar with 5% (v/v) Sheep Blood (Oxoid, Canada) and incubated in 5% CO_2_ at 35˚C for 18-24h.

**Decontamination devices**

Four H_2_O_2_ decontamination systems were evaluated in this study as they had been implemented as part of N95 FFR reprocessing plans at four tertiary care centres across the Greater Toronto Area (Ontario, Canada) during the first COVID-19 pandemic wave. This included the STERRAD 100NX (Advanced Sterilization Products, USA) H_2_O_2_ gas plasma system, the Sterizone VP4 (Stryker Corporation, USA) vaporized H_2_O_2_ and ozone combination system, the ultraviolet light, vaporized H_2_O_2_ and ozone hybrid Clēan Works Clean Flow Mini (Clean Works, Canada) system and the Bioquell Z-2 (ECOLAB, USA) H_2_O_2_ vapor system. Each device was operated according to the manufacturer’s recommended protocols for N95 FFR decontamination where applicable (Table 1). As the Bioquell Z-2 system is an entire room decontamination system, additional experimental parameters were user-defined. The Bioquell Z-2 unit and Bioquell aeration unit were positioned in the centre of a closed room which had high quality epoxy paint on the floors, walls and ceiling. A parametric cycle was selected using a dosing level of 30% H_2_O_2_, a medium load size and room measurements of 7.9m x 5.2m x 2.7m. During Bioquell Z-2 decontamination, the room ventilation was turned off and both exhaust and supply vents and the exterior door were covered or sealed with plastic and tape.

**Respirator inoculation and reprocessing**

Each decontamination device was challenged with up to five different 3M^TM^ N95 FFR models (3M 1860, 1860S, Aura^TM^ 1870+, 8210, 8110S, or VFlex^TM^ 9105S or 1805). Respirators had not been previously decontaminated or worn prior to testing. As this study was performed during the peak of the second pandemic wave in Ontario, each respirator was cut in half vertically using sterile scissors to conserve FFR supplies. For inoculations, three cell suspensions equivalent to a 0.5 McFarland standard (approximately 1.5x10^8^ cells/mL) were prepared for each test organism by emulsifying colonies from fresh subculture plates into sterile 0.45% (v/v) saline. Suspensions were confirmed as 0.5 McFarland equivalents using a VITEK^®^ DensiChek densitometer (bioMérieux Inc, Canada). Each suspension represented a biological replicate. The purity of the bacterial inoculum was confirmed by streaking an aliquot (1 µL) from each suspension onto Columbia Sheep Blood agar and examining colonial morphology after 18-24h of incubation at 35˚C.

Five FFR halves were inoculated for each decontamination system, with three being used as replicates for each test organism, while the others were used as a positive control (untreated) or negative control (inoculated with 0.45% (v/v) saline). Prior to inoculation, three 1 cm x 1 cm squares were drawn onto the patient-facing side of each FFR half using a permanent marker. Each square corresponded to one of three biological replicates and was inoculated with 10 µL of the corresponding 0.5 McFarland suspension, with a sterile applicator stick being used to spread the inoculum evenly on the FFR surface. Inoculated FFR were allowed to air dry for 20-30 minutes before being placed into a sealed Tyvek^®^ pouch for distribution to the testing sites.

Upon receipt of the FFR at each testing site they were processed according to the standard operating procedures for N95 FFR decontamination defined by each site. Decontamination was performed following the manufacturer’s recommendations, with all cycles being evaluated with the use of biological monitors where appropriate. Respirators processed in the STERRAD and Stryker instruments were kept in the original Tyvek^®^ pouches for the duration of the experiment while those from the remaining methods were removed from their packaging and placed on wire racks. After completion of the sterilization process for the Bioquell Z-2 or Clean Works Clean Flow Mini, the FFR were placed back into fresh Tyvek^®^ pouches and returned to the Sinai Health System/University Health Network Microbiology Laboratory (Toronto, ON) within 24 hours. Upon receipt of sterilized FFR in the laboratory, the pouches were opened and the FFR were air dried for 1 hour.

**Determination of decontamination efficacy**

Inoculated segments (1cm^2^ squares) were excised from treated FFR using sterile scissors. Sterile forceps were used to place excised segments into 10 mL of Brain Heart Infusion broth (Oxoid, Canada). To obtain an initial colony count, the inoculated tubes were placed onto an orbital shaker kept at 4˚C for 5 min at 250 rpm and vortexed for 5 seconds. A 1 mL aliquot of each broth tube was flooded onto a Columbia Sheep Blood agar plate (Oxoid, Canada) and incubated for 18-24 hours at 37˚C in 5% CO_2_. The remaining broth was incubated for 48h at 37˚C and sub-cultured to Columbia Sheep Blood agar (Oxoid, Canada) if turbid. The identity of isolates both pre- and post-sterilization (if cultivable) was confirmed using the VITEK^®^ MS MALDI-TOF system (bioMérieux Inc, Canada). Decontamination efficacy (defined as percent reduction in colony forming units (CFU)), was determined by comparing colony counts from segments excised from treated and untreated FFR. Guidance provided to device manufacturers by the US Food and Drug Administration (FDA, 2020) states that a minimum 6-log reduction of two Gram-positive and two Gram-negative vegetative bacteria is needed to demonstrate effectiveness of FFR decontamination.
